# Severe Skin Necrosis and Crusting Following Disseminated Herpes in a Kidney Transplant Patient: A Rare and Alarming Case

**DOI:** 10.1002/kjm2.70062

**Published:** 2025-06-17

**Authors:** Chung‐Ting Cheng, Lee‐Moay Lim, Hung‐Tien Kuo, Yi‐Wen Chiu

**Affiliations:** ^1^ Division of Nephrology, Department of Internal Medicine Kaohsiung Medical University Hospital, Kaohsiung Medical University Kaohsiung Taiwan; ^2^ School of Medicine College of Medicine, Kaohsiung Medical University Kaohsiung Taiwan; ^3^ Graduate Institute of Medicine, College of Medicine, Kaohsiung Medical University Kaohsiung Taiwan

Kidney transplantation significantly enhances survival and quality of life for patients with end‐stage kidney disease (ESKD). However, transplant recipients require lifelong immunosuppression, which increases the risk of infections such as herpes zoster(HZ) [1]. Immunosuppression weakens cellular immunity, leading to severe or atypical presentations of HZ, such as disseminated disease, which has a mortality rate of up to 30% [2]. Here, we present a case of severe disseminated HZ in a kidney transplant recipient.

A 57‐year‐old man who underwent a cadaveric kidney transplant 10 months before this presentation visited our transplant clinic due to diffuse skin ulcers on his right leg. He had previously been treated with Dupilumab for 6 months for atopic dermatitis and was on Tacrolimus, Everolimus, and Myfortic for immunosuppression. He developed generalized vesicles and erythematous papules on his trunk and limbs, which were diagnosed as disseminated HZ. Despite initial treatment with famciclovir 250 mg daily and fusidic acid cream, his skin condition deteriorated, resulting in necrotic ulcers with a secondary bacterial infection.

Physical examination revealed extensive ulcerative lesions on his right leg, characterized by crusting and necrotic plaques and affecting both superficial and deeper layers of the skin (Figure 1A). The lesions had irregular margins and signs of tissue damage. Multiple clusters of crusted vesicles were interspersed with necrotic plaques, distributed throughout the upper, middle, and lower portions of the leg (Figure 1B–D). Laboratory findings showed a hemoglobin level of 8.7 g/dL, a white blood cell count of 6200/μL with 76.6% neutrophils, and a C‐reactive protein level of 118.8 mg/L. Computed tomography of the lower limbs suggested cellulitis with possible superimposed necrotizing fasciitis, and wound cultures subsequently identified *Klebsiella pneumoniae* . He was treated with broad‐spectrum antibiotics, low‐dose Tacrolimus, and steroids, while discontinuing Everolimus and Myfortic after admission.

**FIGURE 1:**
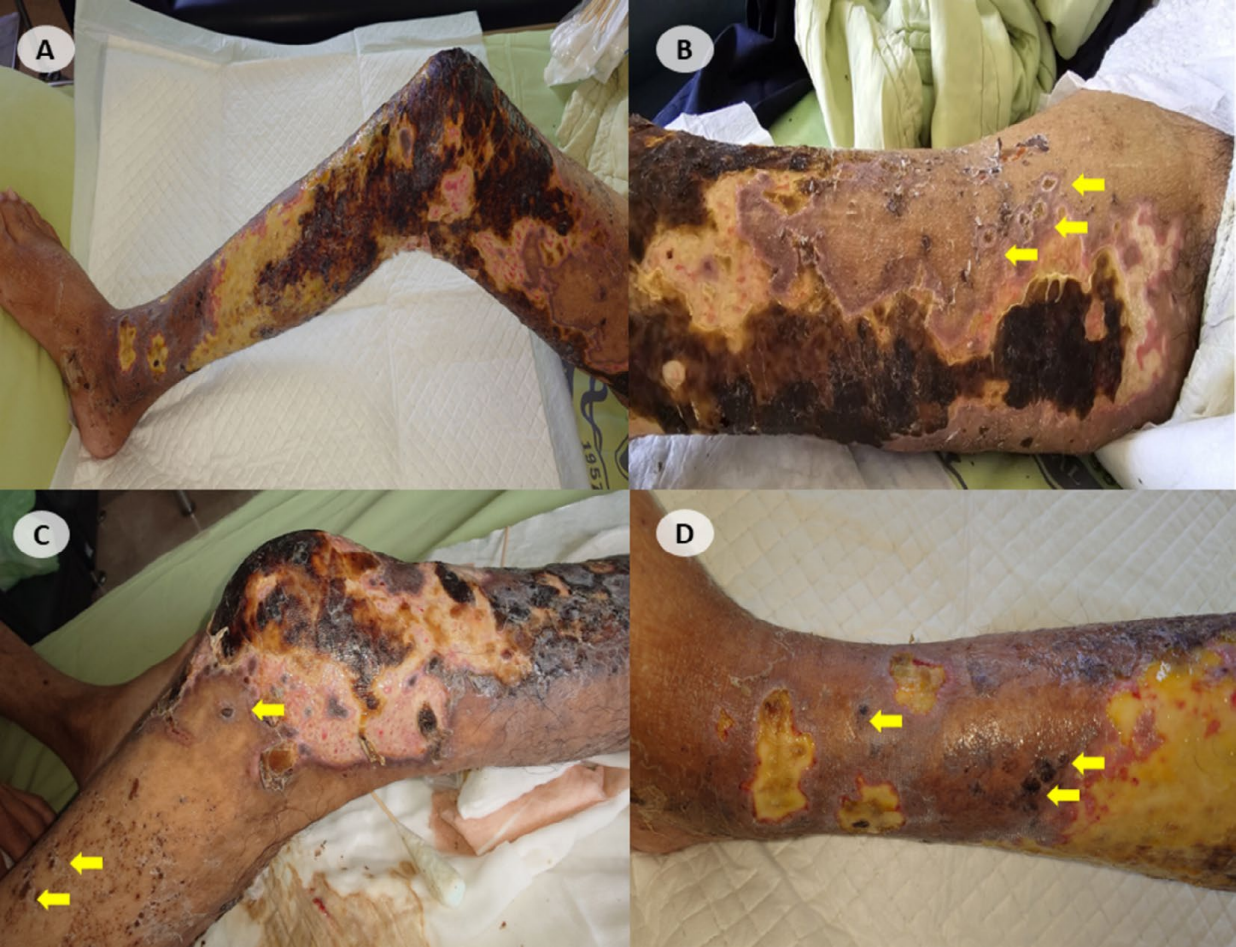
The lesion of the leg appeared as: (A) An extensive ulcerative lesion surrounding the entire leg, characterized by areas of crusting and necrotic plaques. The lesion appeared to involve both superficial and deeper layers of the skin, with irregular margins and signs of tissue damage. There were extensive clusters of crusted vesicles interspersed with necrotic plaques involving: (B) Lesion in the upper leg, (C) Lesions in the middle portion of the leg, (D) Lesion in the lower leg. Arrows show scattered vesicles with crust formation.

During hospitalization, the patient received multidisciplinary care from infectious disease specialists, dermatologists, and plastic surgeons. A skin biopsy was recommended, and antiviral agents for herpes viruses were deemed unnecessary due to the absence of new vesicle formation. Under the adjusted antibiotic regimen, modified immunosuppressive therapy, and consistent wound care, his condition improved. Over a three‐months follow‐up period at the outpatient department, the ulcerative lesions gradually healed with scar formation.

Kidney transplant recipients have a higher incidence of HZ than immunocompetent individuals [2]. Immunosuppressive therapy in kidney transplant recipients leads to significant cellular immune deficiency and minor antibody deficiency, increasing the risk of developing HZ [3]. In a meta‐analysis, Kwon et al. [4] found that solid organ transplant recipients had a higher rate of post‐transplant HZ than the general population and people with chronic medical conditions. In our case, dupilumab therapy aggravated the condition and led to disseminated infection, while the antiproliferative effects of everolimus impaired healing of ruptured vesicles [5]. This case underscores the critical importance of vigilant monitoring and careful evaluation of additional immunomodulatory therapies in kidney transplant recipients. The interaction between immunosuppression and infection control is vital, as immunosuppressive therapies, while necessary to prevent organ rejection, can significantly weaken the immune system and make patients more susceptible to severe infections such as disseminated HZ. Ultimately, this case emphasizes the need for healthcare providers to remain vigilant and proactive in monitoring and managing the delicate balance between immunosuppression and infection control to ensure the well‐being of kidney transplant recipients.

## Conflicts of Interest

The authors declare no conflicts of interest.

## Data Availability

Data sharing not applicable to this article as no datasets were generated or analysed during the current study.
